# Exploiting genetic variation in nitrogen use efficiency for cereal crop improvement

**DOI:** 10.1016/j.pbi.2019.05.003

**Published:** 2019-06

**Authors:** Malcolm J Hawkesford, Simon Griffiths

**Affiliations:** 1 Rothamsted Research, Harpenden, AL5 2JQ, UK; 2 John Innes Centre, Norwich, NR4 7UH, UK

## Abstract

Cereals are the most important sources of calories and nutrition for the human population, and are an essential animal feed. Food security depends on adequate production and demands are predicted to rise as the global population rises. The need for increased yields will have to be coupled to the efficient use of resources including fertilisers such as nitrogen to underpin the sustainability of food production. Although optimally performing crops with high yields require a balanced mineral nutrition, nitrogen fundamentally drives growth and yield as well as requirements for other nutrients. It is estimated that globally only 33% of applied nitrogen fertiliser is recovered in the harvested grain, indicative of a huge waste of resource and potential major pollutant and is thus a major target for crop improvement. Both agronomy and breeding will contribute to improved nitrogen use efficiency (NUE) and an important component of the latter is harnessing germplasm variation. This review will consider the key traits involved in NUE, the potential to exploit genetic variation for these specific traits, and the approaches to be utilised.

## Highlights

• A statement on the critical importance of this area of plant science in terms of food security and sustainability.
• An outline of the critical NUE processes and example studies of identified variation in the complex trait of nitrogen use efficiency.
• Reference to number of critical single key genes/alleles which have major impacts on NUE.
• A description of the most useful genetic approaches being utilised to breed for NUE in cereals.
• Future prospects on the most important areas and approaches likely to have impact.

**Current Opinion in Plant Biology** 2019, **49**:35–42

This review comes from a themed issue on **Physiology and metabolism**

Edited by **Elizabeth A Ainsworth** and **Elizabete Carmo-Silva**

For a complete overview see the Issue and the Editorial

Available online 6th June 2019

**https://doi.org/10.1016/j.pbi.2019.05.003**

1369-5266/© 2019 The Authors. Published by Elsevier Ltd. This is an open access article under the CC BY license (http://creativecommons.org/licenses/by/4.0/).

## Introduction

Cereals including rice, wheat and maize are the most important sources of calories and nutrition for the human population, and in addition they are an essential animal feedstuff. Food security depends on adequate production and demands are predicted to rise in the coming decades as the global population rises. The efficient use of resources including fertilisers such as nitrogen underpins sustainability. Although crops with high yields require a balanced mineral nutrition, nitrogen fundamentally drives growth and yield and requirements for other nutrients. One estimate [1^•^] suggests that globally only 33% of applied nitrogen fertiliser is recovered in the harvested grain. This represents a huge waste of resource and a potential major pollutant through both leaching to water courses and from greenhouse gas emissions, and is thus a major target for crop improvement. More optimistically, as yields are a major target for crop breeding programmes, nitrogen use efficiency (NUE) will also increase in parallel by definition. Indeed in the United Kingdom in recent years, wheat yields have continued to rise modestly, whilst N-inputs have remained constant at the national level, demonstrating improved nitrogen use efficiency [2].

## What is nitrogen use efficiency?

The most fundamental and variable trait influencing NUE is yield; however, the optimum use of nitrogen for crop production may be considered in terms of not only yield but also quality. NUE as a yield efficiency trait is usually defined as yield per unit of available N and is the product of the terms defining uptake efficiency (NUpE) and utilisation efficiency (NUtE), the latter being the effective grain yield produced for the amount of N taken up (see Box 1 and Moll *et al.* [3]). One consequence of this is that high NUE crops will have a high yield but potentially a low N content in the biomass and in the grain. However, additionally NUE for quality must be considered, which requires optimum production of protein the grain and relies both on efficient crop N-uptake and subsequent effective partitioning of nitrogen from vegetative tissues to the grain. High grain protein is often achieved through agronomic intervention of high or additional N applications, often late in the growing season, resulting in a lower grain yield NUE.

Box 1 **Definitions of selected NUE parameters referred to in this article for cereal crops**

**Table:** 

Abbreviation	Trait	Definition	Unit
NUE	Nitrogen use efficiency	Yield (grain) per unit total available nitrogen (fertiliser and mineral N); it is the product of NUpE × NUtE [3]	kg yield/kg N
NUpE	Nitrogen uptake efficiency	Nitrogen taken up by entire above ground biomass as a fraction of total nitrogen available to the crop	kg/kg
NUtE	Nitrogen utilisation efficiency	Yields as a function of the amount of nitrogen taken up	kg/kg
GPC	Grain protein content	The grain protein (content); often the N content (% concentration) × a standard factor to convert to protein (e.g. 5.7)	%
GPD	Grain protein deviation	Actual grain N concentration compared to that expected for a given yield, assuming a linear negative relationship, the residual of an individual point from a regression of grain protein concentration on grain yield [58^•^]	%
NHI	Nitrogen harvest index	The fraction of N in the grain compared to total N taken up, usually at harvest.	Fraction
FRE	Fertiliser recovery efficiency	Grain N from fertiliser as a fraction of that applied as fertiliser: ((N removed in grain) – (N from soil + rain))/fertiliser N applied [1^•^]	%

Alt-text: Box 1

A key measure of nitrogen use efficiency is fertiliser recovery efficiency (FRE) which indicates directly how well applied fertiliser is used and removed by the harvested component of the crop. It is this measure which has been calculated to be as low as 33% in global terms [1^•^].

An alternative metric is how well a crop responds to applied nitrogen. An efficient crop may be one that performs well at low inputs, or alternatively one that performs well at higher inputs producing very high yield at high inputs. The differential yields achieved between low and high inputs may also be a measure of effective fertiliser use and efficient production. Breeding programs have utilised both low and high input systems and even alternated successive generations between the two [4,5].

**Figure 1:**
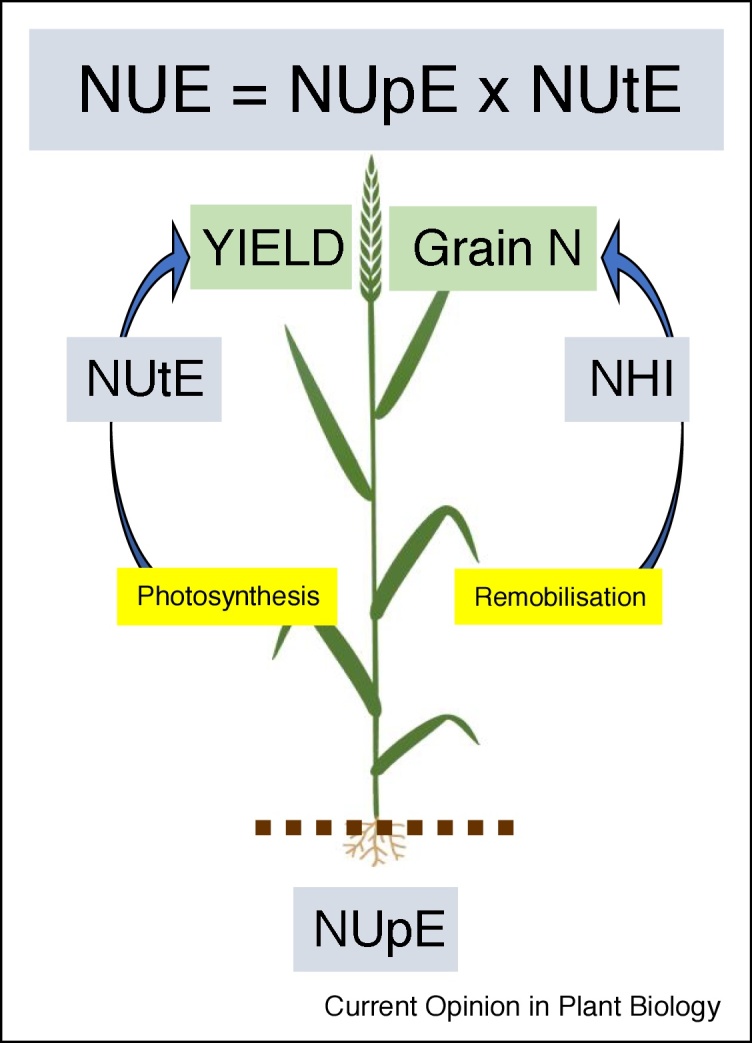
Critical aspects and definitions of NUE as applied to wheat as a model grain crop. The most commonly used definitions as applied to cereal crops are shown in blue. Critical biochemical processes are shown in yellow. The green boxes indicate the final breeding goals of high yield and high grain N (protein) content.

A summary of traits is given in Box 1 and an overview of the interactions between the component traits and key physiological processes contributing to NUE is shown in Figure 1.

## Key traits

A key trait for crop nitrogen use efficiency is the ability of the crop to take up nitrogen: this is a function of the root structure, architecture and function. Subsequent to capture, nitrogen is first utilised to produce an effective canopy. Parameters relating to light capture and photosynthesis will determine yield potential and considerable variation exists and between within any crop species, for example wheat [6]. Secondly the canopy is an essential resource reserve, including of nitrogen which maybe subsequently utilised for grain filling and the importance of individual canopy fractions have been quantified [7]. Canopy height and flowering time affect nitrogen use efficiency [8,9]. This is likely to be due to the above ground biomass affecting the achievable grain yield given favourable harvest index (HI) but may include impacts on root proliferation mediated by *rht* genes and hence effectiveness of the roots in nitrogen acquisition [10].

Yield and quality parameters are usually negatively correlated; however, a trait termed grain protein deviation (GPD) refers to a grain protein content (GPC) greater than expected for any particular yield and is a particularly desirable trait which may be linked to anthesis date and post anthesis N-uptake [11,12] or to grain-specific processes reflected by intrinsic grain gene expression profiles [13]. GPD may be affected by partitioning, as a large fraction of grain N comes from remobilisation from vegetative tissues and is quantified the nitrogen harvest index (NHI; proportion of N in grain as a fraction of the total plant N). Factors influencing nitrogen remobilisation including rates of senescence and transcription factors such as NAM-B1, which influence rates of nutrient remobilisation [14,15,16^••^,^17^]. Whilst most modern hexaploid wheats lack this functional allele, it is present in some Scandinavian populations [18]. Early senescence may enhance N re-use but will have a negative impact of canopy photosynthesis and potential yield. Whilst canopy reserves of resources including N are important for grain filling and show considerable genetic variation [7,19], the senescence of the canopy limits further photosynthesis and reserve accumulation, and ultimately yield. Hence the kinetics of canopy maturation, a highly controlled and regulated process [20], impacts on both final yield and remobilisation efficiency.

Other indicators of nitrogen use efficiency include high yield at low N, particularly important in some subsistence situations where fertilisers may not be readily available. Alternatively, a measure may be responsiveness to applied N, with a desirable trait being a proportionally greater increase with a defined application of N. All of these indicators of efficient use of nitrogen are complex traits involving biochemistry, phenology, architecture and responses to the environment.

## How much genetic variation is there?

There is variation in key NUE traits amongst modern varieties [19,21^•^,^22^,^23^]. However, it is clear that a much greater potential for variation must exist in a wider germplasm base [24^•^]. The key issue with landraces and relatives is that whilst biomass may be high, yields and HI in particular are often very low and the traditional measures of NUE are less useful. However, traits such as total N uptake, N uptake at low availability and biomass potential are all good pointers for useful NUE traits.

The most important architectural influence is stature (height) as influenced by dwarfing genes [8]. The introduction of dwarfing genes not only improves HI and NHI but also decreases susceptibility to lodging at higher nitrogen applications. A potential negative consequence of *rht* genes which whilst decreasing height may also have other pleiotropic consequences such as decreased root proliferation [8,10,25].

Variation in root architecture and function will contribute to the efficiency of uptake. Root proliferation is very dependent upon canopy formation and nitrogen status [26].

A number of studies have dissected root traits as Quantitative Trait Loci (QTL) [10,27], identifying the variation in root proliferation, length, lateral profusion and spread or angle of roots. Many of these studies are laboratory-based due to the difficulties of measurements in the field; this, however, is begging to be resolved with field studies utilising shovelomics [28^•^], root cores or electrophysiological or penetrometer methods for assessing root activity using the proxy of soil drying [29,30].

Mechanisms of nitrate uptake by roots also contribute to uptake efficiency. Large families of genes for nitrate transporters exist in wheat, involved in initial uptake and in internal translocation processes [31] Variation in expression or functioning will impact on nitrate uptake. Specific individual transporter genes have major effects on NUE in rice, for example, *indica* and *japonica* rice may be differentiated by alleles of a low affinity nitrate transporter, NRT1.1.B (OsNPF6.5) [32^••^]. Higher N-uptake and yields and hence NUE in *indica* seem to be specifically dependent on this single allele. In addition, another nitrate transporter, OsNRT2.3, involved in pH sensing in the phloem and which is involved in the regulation of nitrate uptake, shows natural variation within the *indica* subspecies [33].

## Strategies for the identification of genes controlling NUE

**Figure 2:**
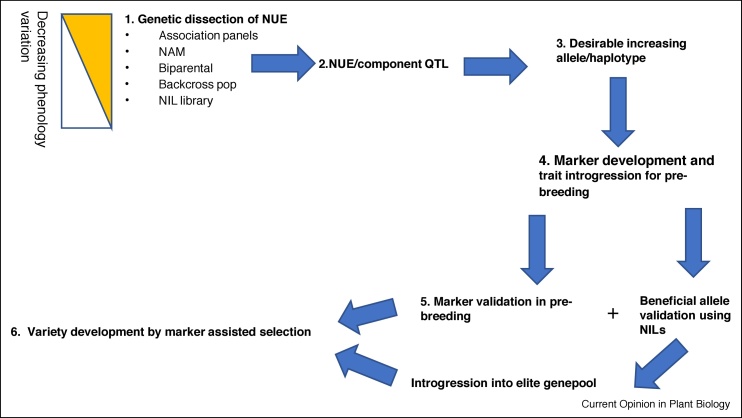
A pipeline for deployment of NUE QTL in breeding is shown. Step 1 shows options for gene discovery and takes account of the potentially confounding effects of variation for phenology in finding useful variation for a given target environment. Step 2 is simple: Were any QTL found?!; in step 3, a judgement is made concerning the use of the increasing allele. This is best done in consultation with end users. In the UK’s current wheat programme (Designing Future Wheat) a committee of academics, genebank managers, pre-breeders, and commercial breeders debate and vote, see http://wisplandracepillar.jic.ac.uk/toolkit.htm. In step 4, the pieces are put in place to move the allele in breeding materials so markers based on the same platform used by breeders are developed (e.g. for wheat single nucleotide-based KASP markers are developed and using highly discriminative alleles across the haplotype derived from high density genotyping or re-sequencing). In the transition from step 4 to 5 the NILs developed are tested in multiple environments to determine if there is an advantage for the NIL compared to the recurrent parent. If the target alleles are already present in elite genepools, the markers can be tested on association panels and within breeding programmes to determine whether they associated with the NUE trait of interest. If the answer to either of these questions is positive, the work moves (in wheat at least) from academic-commercial precompetitive partnership to commercial prebreeding and trait introgression into proprietary germplasm and/or marker are used in established pedigrees.

Figure 2 shows a proposed scheme for the identification of genes controlling NUE and their deployment in breeding. As already discussed, NUE is a highly complex polygenic trait. Consequently, the identification of individual genetic effects requires quantitative genetics approaches and the initial description of such effects is usually as QTL. The past twenty years has seen a widening of the repertoire in statistical methods and population types for the identification of QTL. The optimal approach very much depends on the specific NUE related question being asked. Is the aim to describe genetic mechanisms and trait variation that is already being used in breeding programmes or is the aim to identify new genes/alleles and mechanisms in more diverse germplasm? All the approaches described carry unique strengths and weaknesses.

### Association genetics

Association genetics can be based on panels of genotypes very well adapted to the researchers target environment, sample multiple alleles, and provide very high genetic resolution based on historical recombination events and have been applied in many studies of NUE, for example, in wheat [34]. However, statistical power is relatively low in these materials, a particular problem with NUE traits which tend to be relatively subtle genetic effects displaying low heritability. This problem can be tackled by increasing panel size but this leads to very large and expensive experiments with many NUE traits being expensive to measure (e.g. grinding tissues, CHN analysis). Moreover, association genetics relies on a reasonable balance of alleles at each locus studied, low frequency alleles (typically less than 10%) are eliminated from the analysis. This is a particular problem if rare alleles are the target as would typically be the case when searching for variation in landrace collections and so on. An essential element of successful association studies is the proper consideration of genetic structure. For example, groups of germplasm within a panel may be genetically distinct due to similar pedigree history within each. A NUE trait might be more highly expressed in one group. In this case loci with allele frequency differences between groups will result in false trait marker associations (TMAs) and, equally possible, false negatives.

### QTL mapping using biparental populations

The best-established method of QTL identification is in the use of segregating populations derived from two parents. Fixed homozygous lines are produced by single seed descent (SSD) to produce recombinant inbred lines (RILs) or by the production of doubled haploids. These populations deliver the highest statistical power because only the two parental alleles are segregating at anyone locus and (except in the case of segregation distortion) the population is comprised 50% of each allelic class. Numerous NUE QTL have been identified using this approach for example in rice [35].

### Multiparent populations

Over the last decade attempts have been made to combine the benefits of association and biparental mapping through the use of multiparent populations, most notably nested association mapping (NAM) popularised in maize [36] and multi parent advanced generation intercross (MAGIC) developed in wheat [37]. These allow the simultaneous analysis of multiple alleles and the mapping resolution afforded by recombination captured during the formation of the population as well as historical recombination. The problem of artefactual QTL caused by structure is also removed because the alleles underlying putative TMAs are segregating in the multi parent population. The benefits of these new population types coupled with game changing advances in applied genomics mean that molecular marker data point cost and ability to align to a reference genome is now possible in maize rice and wheat. Work exemplifying this change in the latest of our studies species to achieve post genomic status includes high density marker arrays developed by Winfield *et al.* [38] and, at last, a whole genome sequence assembly [39]. This has led to a step change in the scale of population development with the aim of identifying new and useful genetic variation for complex traits such as NUE, and for example, Wingen *et al.* [40] produced a publicly available NAM population which now represents more than 90 landrace parents and over 10 000 recombinant inbred lines.

### Backcross populations

Backcrossing usually involves a donor parent which carries characteristics of interest and a recurrent parent which is backcrossed with the progeny of the initial cross so that the genomes of the progeny become increasingly (50%) like that of the recurrent parent. After the requisite number of backcrosses, the progeny is then brought to genetic fixation either by single seed descent or doubled haploidy. So each individual of a BC3 (3rd generation of backcrossing) population will be on average 87.5% recurrent parent with 12.5% random segments of the donor parent. After fixation for example by SSD, the donor genome contribution will then be a mere 4.25%. These progenies can be genotyped in each generation and segments selected to tile the entire genome and for a chromosome segment substitution library (CSSL). The populations produced display restricted phenotypic variance, with most individuals almost identical to the recurrent parent. However, lines carrying donor segments that carry QTL influencing NUE only need be compared with the phenotype of the recurrent parent to achieve a highly accurate and precise estimation of the genetic effect from that QTL. Soleimani *et al.* [41] used this approach to dissect phosphorus use in barley.

### Near isogenic lines (NILs)

Empirical selection for agronomic performance, including grain yield has been a good proxy for NUE in wheat [42], rice [43], and maize [44] suggesting that many traits under selection are constitutive and confer benefit at a range of nitrogen availabilities. In these cases, precise genetic stocks developed to study-specific QTL influencing yield and yield components such as those developed by Simmonds *et al.* [45] in wheat, represent a powerful resource for the study of NUE. Chief amongst these are Near Isogenic Lines (NILs) which are produced using the backcrossing approach described above but a single fragment of donor chromosome (genetic foreground) is maintained in the face of backcrossing by active selection of heterozygotes at that locus in each generation either by phenotypic selection where the trait is controlled by a major gene at that locus or, more typically, by marker assisted selection (MAS) using mapped markers that flank the gene of interest or are even within it. NILs are a classic output of advanced QTL studies in which a QTL is validated when the NIL is shown to differ from the recurrent parent in a similar way to the QTL in the original discovery population, that is same direction of effect and similar magnitude. These materials provide an immensely valuable resource for NUE studies. The QTL for the original trait of interest is proven to be robust. Only two lines need to be studies, the NIL and the recurrent parent. This means that multi-level factorial interventions are feasible and deep physiological analysis can be conducted. An increasing pool of NIL resources are now available and their specific use in NUE studies has been carried out in wheat for example by Kowalski *et al.* [46] and whole libraries of NILs representing all major agronomic QTL identified so far from well studies reference populations such as Avalon × Cadenza [47] surely present a rich seam of discovery for future NUE research.

## The impact of phenology on meaningful gene discovery

The word phenology was coined by 19th century English naturalists recording the timing of key natural history events within each season such as the appearance of blossom on trees or frog spawn in the local pond [48]. In reference to crops it describes the timing of developmental transitions such as the transition from vegetative to reproductive growth, the onset of stem extension, the emergence of the inflorescence, anthesis, senescence, and maturity. Phenology is genetically determined with a number of environmental cues modifying the crop response. Optimisation of the crop phenological profile is the major driver of crop adaptation. So, it comes as little surprise that phenology is shown to correlate with NUE is almost every study in which it is measured and QTL for phenology traits often collocate with NUE and it is components, most likely through pleiotropy. This raises a very important question for NUE gene discovery studies. For example, if a major phenology QTL such as *Ppd-1* controlling photoperiod sensitivity in wheat, or Hd1 in rice, and so on is segregating and the allele which is associated with poor adaptation to that environment is also the low allele for NUE in that population has anything useful been learnt about NUE in that scenario? The same can be said about major genes controlling crop height. In most cases the answer is no, and researchers do not pursue these effects. The segregation of the effect still causes a problem though, because it itself it increases the noise to signal ratio for other NUE loci (often of smaller effect) segregating in the same population which are of greater interest but might not be detected at all in this scenario.

The populations described above were described in order of decreasing phenology variance. In wheat for example many association panels might exhibit a range of flowering times up to one month whereas a NIL will usually have the same phenological profile as the recurrent parent (unless the foreground selection was for a phenology QTL). All the better if the recurrent parent is well adapted to the growing environment. This means that any effect detected can be attributed to NUE *per se*, and not as a relatively simple and low value secondary consequence of improved adaptation. This is not to say that the fine tuning of phenology does not play an important role in the maximisation of NUE. Within genotypes that are equally well adapted improvements in NUE can be achieved by fine tuning phenological patterns such as the rate of floret abortion [49].

Using NILs does not fully answer the phenology problem as *de novo* NUE QTL needs to be conducted. Backcross and CSSL populations are a powerful way to achieve this but a large number of lines need to be studied in order to scan a single genome. Multiparent populations provide an exciting alternative. Although they often comprise 100 s or even 1000 s of individuals, subsamples of these populations still provides an efficient strategy for QTL discovery. If phenology data are available for the whole population these selections can be made on the basis of a narrow phenological window for any given environment, thus minimising these confounding effects in the primary phase of NUE QTL detection.

## Prospects for crop improvement

The major target for breeders and growers is high yield. Specifically, in the case of wheat, a high final protein content is desirable for bread-making quality. For all cereal crops and agronomic systems, the key priority is efficient capture of N by the root systems, to improve on the low worldwide efficiency [1^•^]. Appropriate agronomy will contribute greatly to this; however, genetic approaches have not fully targeted root traits and there is likely scope for improvement. An efficient canopy, targeting both structural and photosynthetic traits will improve yield and NUE as defined as nitrogen requirement; however, this may be at the expense of quality. Possibly the most important target will be to improve the N use efficiency at higher N inputs, particularly by ensuring efficiency of use of the higher N inputs in the grain.

**Figure 3:**
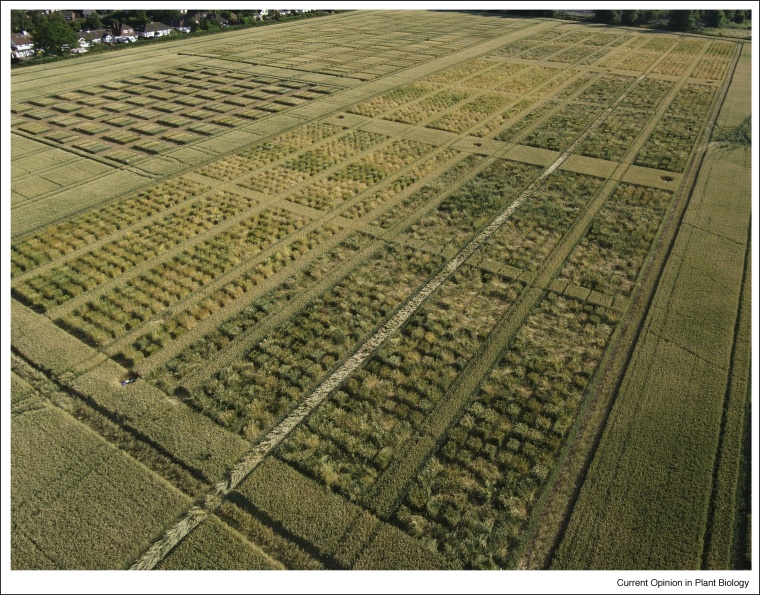
A field trial of diverse wheat accessions growing at two nitrogen fertiliser input rates (the right-hand blocks have the highest N-inputs, a consequence of which is the delayed senescence and observed lodging. The trial was located at Rothamsted in the UK in 2017. Germplasm comprises crosses of Watkins accessions with Paragon as a common parent. Huge differences in form and phenology contribute to differing NUE parameters.

A hugely useful resource is the wide genetic variation available to cereal breeders and researchers. Significant but limited variation exists in modern germplasm pools; however, a huge opportunity exists to exploit more diverse germplasm collections such as the Watkins collection [50] and identify efficiency alleles which may have been lost to the modern germplasm pool. Screening and exploiting such material require a major commitment to large scale trials at contrasting locations and in multiple years, as well as the expertise to phenotype for phenology, architecture and nutrient use efficiency parameters. A typical trial of bi-parental wheat populations illustrates the diversity of form and the impact of nitrogen inputs (Figure 3). The critical challenge is having the right phenotypic screening strategies which can be applied with high throughput at the appropriate scale. The use of optical sensors [51] and crop indices such NDVI (normalised difference vegetation index) [52] to measure canopy development and canopy nutritional status have been widely employed, both with ground-based observations using spectrometers and hand-held contact devices such as SPAD meters [53] or using aerial imagery [54,55]. The most recent developments include automated mobile [56] and fixed [57] platforms for high temporal and spatial analysis of such parameters.

## Conflict of interest statement

Nothing declared.

## References and recommended reading

Papers of particular interest, published within the period of review, have been highlighted as:

• of special interest
•• of outstanding interest
